# Association of Allergic Symptoms in the First 2 Years of Life With Sleep Outcomes Among Chinese Toddlers

**DOI:** 10.3389/fped.2021.791369

**Published:** 2022-01-12

**Authors:** Yujing Chen, Lizi Lin, Bin Hong, Shamshad Karatela, Wenting Pan, Shengchi Wu, Nu Tang, Yuxuan Wang, Jin Jing, Li Cai

**Affiliations:** ^1^Department of Maternal and Child Health, School of Public Health, Sun Yat-sen University, Guangzhou, China; ^2^Department of Occupational and Environmental Health, School of Public Health, Sun Yat-sen University, Guangzhou, China; ^3^Department of Health Care, Maternal and Child Health Care Hospital of Yuexiu District, Guangzhou, China; ^4^Faculty of Health and Behavioural Sciences, Pharmacy Australia Centre of Excellence, University of Queensland, Woolloongabba, QLD, Australia; ^5^Institute of Tropical Health and Medicine (AITHM), James Cook University, Townsville, QLD, Australia; ^6^Quality and Safety Management Office, The Second Affiliated Hospital of Guangxi Medical University, Nanning, China; ^7^Department of Health Care, Foshan Women and Children Hospital, Foshan, China; ^8^Global Health Research Center, Duke Kunshan University, Kunshan, China

**Keywords:** allergic symptoms, allergic disease, sleep disturbance, sleep duration, toddler

## Abstract

**Background:** Previous studies have linked allergic symptoms to sleep in children, but the associations might be different when considering different types of allergic symptoms or sleep outcomes. Moreover, the combined effects of multiple allergic symptoms remain unclear in early life. This study aimed to investigate the associations between multiple allergic symptoms and sleep outcomes in early life.

**Methods:** We included 673 toddlers aged 2 years from a birth cohort in Guangzhou, China. We identified allergic symptoms (skin, eyes and nose, gastrointestinal tract, mouth and lips, and wheeze) within 2 years via standard questionnaires. Sleep outcomes including sleep duration and quality over the past month were assessed based on the Chinese version of the Brief Infant Sleep Questionnaire. Associations between allergic symptoms and sleep outcomes were examined using multivariable linear regression and logistic regression.

**Results:** Compared to children without allergic symptoms, children with allergic nasal and ocular symptoms had higher odds of frequent nighttime awakenings (OR = 1.41; 95% CI: 1.03, 1.93) and irregular sleep (OR = 1.45, 95% CI: 1.05, 2.00); children with allergic gastrointestinal symptoms slept 0.28 h less during nighttime (95% CI: −0.48, −0.07) and 0.25 h less per day (95% CI: −0.43, −0.08), and had 59% higher odds of irregular sleep (95% CI: 1.24, 2.04). We also found significant association of multiple allergic symptoms with shortened nighttime sleep duration and increased irregular sleep. Whereas, allergic skin, mouth and lips, and wheeze symptoms were not significantly associated with sleep outcomes.

**Conclusion:** Allergic symptoms within 2 years of age were adversely associated with sleep outcomes, which highlight the importance of early screening of allergic symptoms in toddlers in order to improve their sleep outcomes.

## Introduction

Sleep plays a critical role in promoting child health and development (1). Short sleep duration and apparent epidemic of sleep disturbances (e.g., 20–35% for difficult falling or maintaining sleep) among infants and toddlers have posed a great health concern worldwide (2, 3). Insufficient or low-quality sleep during early life has been linked to myriad of negative health consequences, including impaired neurocognitive function, emotional disorder, metabolic and cardiovascular diseases, etc. (4, 5). Therefore, developing early-life intervention strategies targeting modifiable risk factors for sleep issues is imperative. In addition to environmental, behavioral and social factors that potentially impact sleep (6), current studies have linked sleep outcomes to allergic diseases and related symptoms in toddlers, which has been increasingly prevalent worldwide.

Allergic diseases were often under-diagnosed and untreated in the early life despite allergic symptoms appearing in infancy (7, 8). Toddlers might present allergy-related signs/symptoms in specific organs or locations of the body, such as the skin, upper and lower airway, and digestive system (9). In Chinese cities, 41% of infants and toddlers have ever experienced one or more allergic symptoms (10). These bothersome allergic symptoms tend to deteriorate at night and may interfere with sleep process through pathophysiologic mechanisms of fluctuations in inflammatory mediators, melatonin and cortisol dysregulation, and a broader autonomic dysfunction (11–13).

Most of the previous epidemiological studies have evaluated allergic symptoms in relation to sleep among school-aged children and adolescents (14–16). However, there has been minimal attention to infants and toddlers with inconsistent results when considering different aspects of sleep outcomes. For example, a cross-sectional study of 4,085 Thai infants indicated that severe symptoms of atopic dermatitis (AD) were associated with shorter sleep duration instead of number of night wakings and difficulty falling asleep (17). Whereas, one case-control study in Turkey (18) and one cross-sectional study in the USA (19) suggested that young children with AD and asthma had similar sleep duration but frequent night waking compared with their counterparts. However, these studies have generally examined the allergy-related sleep disturbances and have only focused on one specific allergic condition or symptoms. To our knowledge, no other studies have included measures of multiple comorbid allergic symptoms to investigate their associations with sleep during early life.

Therefore, the present study aimed to investigate the associations between multiple allergic symptoms and sleep outcomes in toddlers aged 2 years. Using the data from a birth cohort, we hypothesized that the associations might be different when considering different allergic symptoms and sleep outcomes, and toddlers with multiple allergic symptoms might have the worst sleep outcomes compared to their counterparts.

## Materials and Methods

### Study Population

The study subjects were mother-child pairs from a birth cohort study (Registration number: NCT03023293) in Guangzhou, China. We recruited pregnant women between 20 and 28 weeks of gestation to whom face-to-face interviews were conducted at Yuexiu district maternal and child health hospital during 2017–2018, as described in detail elsewhere (20). The mother-infant pairs were invited for a series of follow-up visits which included clinical examinations and telephone interviews at the postnatal 6 weeks, 6 months, and 2 years. This study enrolled 706 toddlers at 2 years of age. Toddlers were excluded if they had serious complications at birth (*n* = 11), or had missing information on allergy symptoms (*n* = 8) or sleep (*n* = 14). Thus, after the exclusion criteria, 673 children were included in the final analysis. The ethics committee of the School of Public Health of Sun Yat-sen University approved the study protocol, and all participants provided written informed consent before inclusion.

### Measurements of Allergic Symptoms

Allergic symptoms were classified according to the affected organ systems and measured with standardized questions adapted from the International Studies on Asthma and Allergies in Childhood (ISAAC) questionnaire (21). For each classification of allergic symptoms, we determined the specific manifestations in conformity to a guideline released by Subspecialty Group of Immunology, Society of Pediatrics, Chinese Medical Association (22). Therefore, five types of parent-reported allergic symptoms were assessed as follows: (a) Allergic skin symptoms were defined based on an affirmative answer to the question, “Has your child ever had an itchy rash in the joints and creases of his body?” or “Has your child ever had a nettle rash which are short lived, disappearing within a few days?”; (b) Allergic nasal and ocular symptoms were defined based on an affirmative answer to the question, “Has your child ever had a problem with repeated sneezing, a runny, or a blocked nose when he/she did not have a cold or the flu, persisting for at least 1 h per day, and 2 days per week?” or “has this nose problem been accompanied by itchy, red or watery eyes?”; (c) Allergic gastrointestinal (GI) symptoms were defined based on an affirmative answer to the question, “Has your child ever had frequent vomiting, abdominal pain, diarrhea, loose or bloody stools, constipation, or excessive gas exhaust, persisting for at least 3 days per week?”; (d) Allergic mouth and lips symptoms were defined based on an affirmative answer to the question, “Has your child ever had an edema or an itch around the lips or mouth?”; and (e) Wheeze was defined based on an affirmative answer to the question, “Has your child ever had wheezing or whistling in the chest, but not noisy breathing from the nose?”

We collected information regarding the age at which allergic symptoms first occurred and relapsed from birth to 2 years of age, and different types of allergic symptoms were binary variables encoded as 0 for no and 1 for yes. We further grouped allergic symptoms to define allergic multimorbidity into 3 categories: none, single symptom, and multiple symptoms.

We also collected information regarding the physician-diagnosed allergic diseases, including eczema, AD, urticaria, food allergy, allergic rhinitis (AR), hay fever, allergic conjunctivitis, and asthma.

### Measurements of Sleep Outcomes

Sleep outcomes including sleep duration and quality were assessed at 2 years of age based on the Chinese version of Brief Infant Sleep Questionnaire (BISQ), recommended by the national “Guideline for sleep hygiene among children aged 0–5 years” (23, 24). The BISQ has established sensitivity in detecting developmental sleep pattern in infants and has been validated against actigraphy and sleep diaries (24). Parents were asked to report their children's sleep outcomes over the past month, including average bedtime, wake-up time, sleep onset latency (SOL, how long it took to fall asleep), frequency of nighttime awakenings (nights per week), duration of nighttime awakenings per night (22:00 p.m.−6:00 a.m.), daytime sleep duration, and perception on child's irregular sleep (yes or no). We also collected information on children's sleep hygiene, including how to fall asleep (in bed alone, yes or no) and bed sharing with family members (yes or no). Nighttime sleep duration was calculated on the basis of bedtime, SOL and wake-up time. Total sleep duration was calculated by summing up nighttime and daytime sleep duration. Difficulty falling asleep (defined as SOL > 20 min) (23), frequent nighttime awakenings (defined as waking up for 3 or more nights per week) (25), and parent-perceived irregular sleep were considered as measurements of sleep-quality disturbances in this study.

### Assessments of Covariates

We collected maternal socio-demographic characteristics via personal interview during the baseline investigation, including maternal age, maternal highest education level, and monthly household income. Information on delivery mode, preterm birth (gestational age <37 weeks) and child's gender were obtained from the hospital birth registry system. We also collected other related information via parent-reported questionnaires at the age of 6 months and 2 years, including exclusive breastfeeding duration, family history of allergy, and household secondhand smoke (family members who lived with the child smoked in presence of the child). Data on child's weight and height at 2 years of age was extracted from the hospital medical system, and body mass index (BMI) Z-score was calculated based on the World Health Organization (WHO) Growth Standards.

### Statistical Analysis

Demographic characteristics, sleep outcomes, and allergic symptoms of study population were described as *mean* ± *SD* or percentages. To compare the difference of sleep outcomes by specific allergic symptoms and allergic multimorbidity, *t*-test or ANOVA was conducted for continuous variables and chi-square test for categorical variables.

Multivariable linear regression was used to estimate the associations between allergic symptoms and sleep duration (total, nighttime, and daytime sleep duration), and logistic regression was utilized to evaluate the odds ratios (ORs) of sleep-quality disturbances (difficulty falling asleep, frequent nighttime awakenings, and irregular sleep). The five types of allergic symptoms and allergic multimorbidity were examined in separate regression models, respectively. In all regressions, main model was adjusted for child's gender, maternal age at enrollment, maternal education level, monthly household income, family history of allergy, delivery mode, and household secondhand smoke.

We further conducted several sensitivity analyses. We identified additional potential risk factors (i.e., preterm birth, duration of exclusive breastfeeding, BMI Z-score, and sleep hygiene factors) and added each of them to our main regression models, respectively. We also categorized total sleep duration as insufficient sleep (<11 h, yes or no) (23) and assessed difficulty falling asleep using an elevated cutoff (SOL >30 min) (2) to examine whether the results were consistent.

Missing values in covariates were handled by multiple imputations using a fully conditional specification (FCS) method which leads to satisfactory results with five iterations (26). All analyses were performed in the SAS 9.4 (SAS Institute, Inc., Cary, NC, USA). Two-sided values of *P* < 0.05 were considered statistically significant.

## Results

### Demographic Characteristics and Sleep Outcomes

As shown in Table 1, the study sample was composed of 673 toddlers aged 2 years, of whom 51.13% were boys and 29.87% had a family history of allergy. On average, these children slept for 11.74 ± 1.06 h per day including 9.51 ± 1.01 h during nighttime. About 61.66% of the toddlers had difficulty in falling asleep (SOL > 20 min), while 15.01% of them woke up for 3 or more nights per week, and 13.67% had parent-perceived irregular sleep.

**Table 1:** Demographic characteristics and sleep outcomes among 673 toddlers aged 2 years.

**Characteristics**	**All participants**
**Household**	
Maternal age>35 years, *n* (%)	117 (17.38)
**Maternal education level**, ***n*** **(%)**	
Senior high school and below	214 (34.41)
Junior college	200 (32.15)
University and above	208 (33.44)
**Monthly household income**, ***n*** **(%)**	
<4,000 RMB	122 (19.68)
4,000–6,000 RMB	137 (22.10)
6,000–10,000 RMB	164 (26.45)
≥10,000 RMB	197 (31.77)
**Child**	
Male, *n* (%)	318 (51.13)
Family history of allergy, *n* (%)	201 (29.87)
Household secondhand smoke, *n* (%)	89 (13.22)
**Exclusive breastfeeding duration**, ***n*** **(%)**	
0 ~ <4 months	236 (44.95)
4 ~ <6 months	100 (19.05)
≥6 months	189 (36.00)
**Delivery mode**, ***n*** **(%)**	
Vaginal delivery	332 (58.55)
Cesarean section	223 (39.33)
Other mode	12 (2.12)
Preterm birth, *n* (%)	19 (3.35)
BMI Z-score, *mean ± SD*	−0.24 ± 1.22
**Sleep outcomes**	
Falling asleep in bed alone, *n* (%)	55 (8.17)
Bed sharing with family members, *n* (%)	619 (91.98)
Bedtime (hh:mm), *mean ± SD*	21:45 ± 2:18
Wake-up time (hh:mm), *mean ± SD*	8:05 ± 1:05
Nighttime sleep duration (h), *mean ± SD*	9.61 ± 0.91
Daytime sleep duration (h), *mean ± SD*	2.13 ± 0.67
Total sleep duration (h), *mean ± SD*	11.74 ± 1.06
Insufficient sleep ^a^, *n* (%)	127 (18.87)
Sleep onset latency (min), *mean ± SD*	27.84 ± 18.37
Difficulty falling asleep ^b^, *n* (%)	415 (61.66)
Frequent nighttime awakenings ^c^, *n* (%)	101 (15.01)
Duration of nighttime awakenings (min), *mean ± SD*	3.24 ± 10.98
Parent-perceived irregular sleep, *n* (%)	92 (13.67)

*Data presented are n (%) or mean ± SD. BMI, Body mass index*.

a *Insufficient sleep: total sleep duration <11 h*.

b *Difficulty falling asleep: sleep onset latency > 20 min*.

c *Frequent nighttime awakenings: ≥3 nights/week*.

### Allergic Symptoms

**Figure 1:**
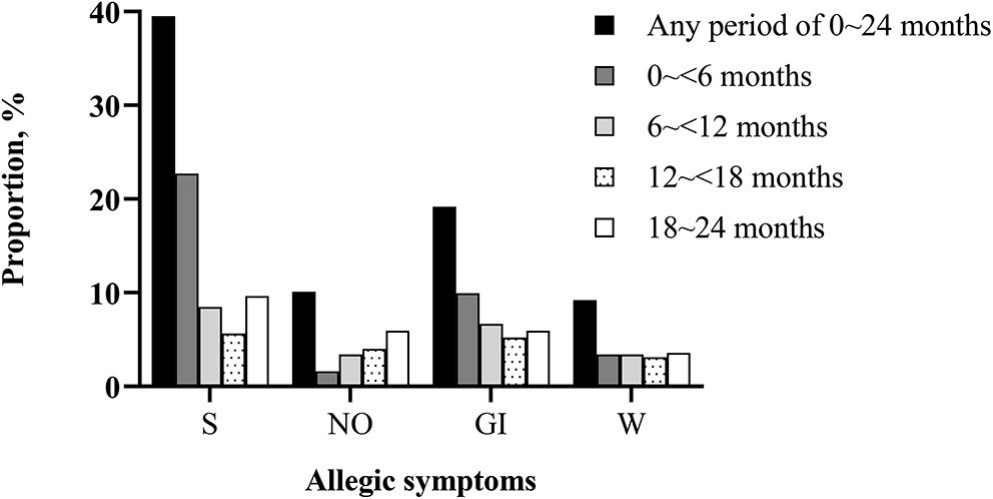
Proportion of toddlers with allergic symptoms in different periods of the first 2 years of life (*N* = 673). S, skin symptoms; NO, nasal and ocular symptoms; GI, gastrointestinal symptoms; W, wheeze. There were 37 (5.50%) children ever had mouth and lips symptoms during the first 2 years of life (data not shown).

As shown in Figure 1, the proportion of having allergic symptoms in the first 2 years of life were highest in the skin (39.52%) and the lowest in the month and lips (5.50%). For allergic multimorbidity, there were 379 (56.32%) toddlers with any allergic symptoms, of whom 137 had multiple allergic symptoms. Details of the comparisons between physician-diagnosed allergic diseases and allergic symptoms were presented in Supplementary Figure 1.

### Associations of Different Allergic Symptoms With Sleep Outcomes

Table 2 summarized the associations of allergic symptoms with sleep duration. Compared with children without GI symptoms, children with GI symptoms had shorter total sleep duration (β = −0.28, 95% CI: −0.48, −0.07) and nighttime sleep duration (β = −0.25, 95% CI: −0.43, −0.08). Multiple allergic symptoms were inversely associated with nighttime sleep duration (β = −0.21, 95% CI: −0.39, −0.02). There were no significant associations between other allergic symptoms and sleep duration.

**Table 2:** Association of allergic symptoms in the first 2 years with sleep duration at the 2 years of age (*N* = 673).

**Allergic symptoms^c^**	**Total sleep duration**		**Nighttime sleep duration**		**Daytime sleep duration**	
	***Mean*±*SD*^a^, h**	**Adjusted β (95% CI)^b^**	***Mean*±*SD*^a^, h**	**Adjusted β (95% CI)^b^**	***Mean*±*SD*^a^, h**	**Adjusted β (95% CI)^b^**
**Skin symptoms**						
Never had	11.77 ± 1.06	0.00 (ref)	9.65 ± 0.91	0.00 (ref)	2.12 ± 0.66	0.00 (ref)
Ever had	11.70 ± 1.07	−0.07 (−0.23, 0.09)	9.56 ± 0.92	−0.07 (−0.21, 0.07)	2.14 ± 0.70	0.00 (−0.10, 0.10)
**Nasal and ocular symptoms**						
Never had	11.73 ± 1.07	0.00 (ref)	9.62 ± 0.93	0.00 (ref)	**2.11 ± 0.67**	0.00 (ref)
Ever had	11.83 ± 1.00	0.10 (−0.17, 0.37)	9.54 ± 0.80	−0.04 (−0.27, 0.19)	**2.28 ± 0.71**	0.14 (−0.03, 0.31)
**Gastrointestinal symptoms**						
Never had	**11.79 ± 1.04**	0.00 (ref)	**9.66 ± 0.89**	0.00 (ref)	2.13 ± 0.66	0.00 (ref)
Ever had	**11.52 ± 1.10**	**−0.28 (−0.48**, **−0.07)**	**9.42 ± 0.98**	**−0.25 (−0.43**, **−0.08)**	2.10 ± 0.74	−0.02 (−0.15, 0.10)
**Wheeze**						
Never had	11.73 ± 1.07	0.00 (ref)	9.62 ± 0.92	0.00 (ref)	2.11 ± 0.68	0.00 (ref)
Ever had	11.88 ± 0.93	0.13 (−0.15, 0.41)	9.60 ± 0.83	0.00 (−0.24, 0.24)	2.28 ± 0.56	0.13 (−0.05, 0.30)
**Allergic multimorbidity**						
None	11.80 ± 1.01	0.00 (ref)	**9.71 ± 0.89**	0.00 (ref)	2.09 ± 0.64	0.00 (ref)
Single symptom	11.72 ± 1.13	−0.07 (−0.25, 0.11)	**9.58 ± 0.94**	−0.11 (−0.27, 0.04)	2.14 ± 0.69	0.04 (−0.07, 0.15)
Multiple symptoms	11.64 ± 1.04	−0.16 (−0.38, 0.05)	**9.47 ± 0.91**	**−0.21 (−0.39**, **−0.02)**	2.17 ± 0.70	0.04 (−0.09, 0.18)
*P*_allergic multimorbidity_	0.130	0.135	**0.010**	**0.024**	0.275	0.424

a *Difference of mean between groups using T-test or ANOVA*.

b *Adjusted estimated coefficient (β) and 95% confidence interval (CI) of multivariable linear regression model adjusted for child's gender, maternal age at enrollment, maternal education level, monthly household income, family history of allergy, delivery mode, household secondhand smoke*.

*Statistically significant results are in bold (P < 0.05)*.

*Missing values in covariates were handled by multiple imputation (imputation = 5)*.

c *Mouth and lips symptoms were not significantly associated with total, nighttime, and daytime sleep duration (data not shown)*.

The associations between allergic symptoms and sleep-quality disturbances were shown in Table 3. Children with allergic nasal and ocular symptoms had 41% higher risk of frequent nighttime awakening (95% CI: 1.03, 1.93), compared with those without allergic nasal and ocular symptoms. Allergic GI symptoms (adjusted OR [aOR ] = 1.59; 95% CI: 1.24, 2.04), nasal and ocular symptoms (aOR = 1.45; 95% CI: 1.05, 2.00), and multiple symptoms (aOR = 2.56; 95% CI: 1.42, 4.62) were associated with greater risk of parent-perceived irregular sleep. Non-significant associations were observed between any allergic symptoms and difficulty falling asleep. Allergic skin symptoms, wheeze, and mouth and lips symptoms were not statistically associated with any measurements of sleep disturbances.

**Table 3:** Association of allergic symptoms in the first 2 years with sleep-quality disturbances at 2 years of age (*N* = 673).

**Allergic symptoms^c^**	**Difficulty falling asleep**		**Frequent nighttime awakening**		**Irregular sleep**	
	***n* (*%*)^a^**	**Adjusted OR (95% CI)^b^**	***n* (*%*)^a^**	**Adjusted OR (95% CI)^b^**	***n* (*%*)^a^**	**Adjusted OR (95% CI)^b^**
**Skin symptoms**							
Never had	246 (60.44)	1.00 (ref)	60 (14.74)	1.00 (ref)	54 (13.27)	1.00 (ref)
Ever had	169 (63.53)	1.05 (0.89, 1.24)	41 (39.52)	1.05 (0.84, 1.31)	38 (14.29)	1.08 (0.85, 1.36)
**Nasal and ocular symptoms**							
Never had	332 (61.03)	1.00 (ref)	**85 (14.05)**	1.00 (ref)	**76 (12.56)**	1.00 (ref)
Ever had	83 (64.34)	1.07 (0.87, 1.31)	**16 (23.53)**	**1.41 (1.03, 1.93)**	**16 (23.35)**	**1.45 (1.05, 2.00)**
**Gastrointestinal symptoms**							
Never had	372 (61.49)	1.00 (ref)	80 (14.71)	1.00 (ref)	**61 (11.21)**	1.00 (ref)
Ever had	43 (63.24)	1.05 (0.89, 1.24)	21 (16.28)	1.06 (0.81, 1.39)	**31 (24.03)**	**1.59 (1.24, 2.04)**
**Wheeze**							
Never had	376 (61.54)	1.00 (ref)	92 (15.06)	1.00 (ref)	79 (12.93)	1.00 (ref)
Ever had	39 (62.90)	0.97 (1.15, 0.73)	90 (14.52)	0.97 (0.66, 1.42)	13 (20.97)	1.27 (0.89, 1.80)
**Allergic multimorbidity**							
None	177 (60.20)	1.00 (ref)	40 (13.61)	1.00 (ref)	**29 (9.86)**	1.00 (ref)
Single symptom	145 (59.52)	0.96 (0.68, 1.38)	37 (15.29)	1.15 (0.71, 1.86)	**34 (14.05)**	1.52 (0.88, 2.61)
Multiple symptoms	93 (67.88)	1.31 (0.84, 2.03)	24 (17.52)	1.35 (0.78, 2.34)	**29 (21.17)**	**2.56 (1.42, 4.62)**
*P*_allergic multimorbidity_	0.135	0.313	0.287	0.287	**0.002**	**0.002**

a *Proportion of toddlers with sleep disturbances; difference of proportion between groups using Chi-square test*.

b *Adjusted odd ratio (OR) and 95% confidence interval (CI) of logistic regression model adjusted for child's gender, maternal age at enrollment, maternal education level, monthly household income, family history of allergy, delivery mode, household secondhand smoke*.

*Statistically significant results (P < 0.05) are in bold*.

*Missing values in covariates were handled by multiple imputation (imputation = 5)*.

c *Mouth and lips symptoms were not significantly associated with difficulty falling asleep, frequent nighttime awakening, and irregular sleep (data not shown)*.

### Sensitivity Analyses

Sensitivity analyses indicated that the results were comparable when we additionally adjusted for several important risk factors (Supplementary Tables 1, 2). The results remained similar when using binary outcomes of insufficient sleep (<11 h) and elevated cutoff value (SOL > 30 min) for difficulty falling asleep (Supplementary Tables 3, 4).

## Discussion

In the present study, we confirmed that the allergic symptoms and sleep disturbances were highly prevalent among toddlers as reported previously (2, 10). We found that the associations between allergic symptoms and poor sleep outcomes were stronger in 2-year-old toddlers with several allergic symptoms that appeared in nose, eyes and digestive system. We also found that toddlers with multiple allergic symptoms had shorter nighttime sleep duration and higher risk of parent-reported irregular sleep. To our knowledge, our study is the first of its kind to have identified multiple allergic symptoms and different sleep outcomes among toddlers, in a population with substantial under-diagnosed allergic conditions.

Our results indicated that toddlers with allergic nasal and ocular symptoms had higher risk of frequent nighttime awakening and parent-perceived irregular sleep, which were consistent with the results of two cross-sectional studies conducted in Spain and the USA (14, 27). These studies have found significant associations between allergic nasal symptoms and higher risk of night awakening in adolescents, and we have extended similar findings in toddlers. Mechanistically, circadian fluctuations in inflammatory cytokines and mediators may be responsible for exacerbating airway obstruction during the night (12), and some of these inflammatory mediators (e.g., histamine, cysteinyl leukotriene, and interleukin-6) also participate in the regulation of sleep-wake cycle and circadian rhythm, resulting in frequent arousals and sleep disturbances (13, 28). Intervention studies has indicated that anti-inflammatory therapy to reduce AR symptoms led to improved sleep of adolescents and adults (29, 30), demonstrating that early interventions also should be developed for toddlers in the future.

We also found that toddlers with allergic GI symptoms slept 0.28 h less per day (mainly at night) and had higher risk of total sleep duration less than the recommended 11–14 h (23). GI allergy in infancy is usually triggered by food allergens, which was associated with frequent nighttime awakening in a case-control study conducted in UK (31). An earlier study conducted in Belgium also found that persistent sleeplessness in infants and toddlers was attributed to an undiagnosed intolerance to cow's milk (32). When focusing on diagnosed food allergy, two similarly designed studies conducted in Turkey and Poland (33, 34) observed the adverse association between childhood food allergy and sleep quality, whereas the role of GI manifestations of food allergy in this relationship was not clarified. A strength of our study is to provide specific evidence on allergic GI symptoms in relation to reduced sleep duration among toddlers. This finding might be partly explained by the later bedtime (22:13 ± 1:07 vs. 21:57 ± 0:54, *P* < 0.05) and longer SOL (*P* = 0.076) in allergic GI symptoms group. It has been demonstrated that infants who had earlier sleep onset also slept longer during the nighttime (35), and 1 h later in bedtime was linked to a decrease of ~30 min in sleep duration at night (36), lending support to our finding. Imbalance of the autonomic system might underly the pathophysiology of allergic GI symptoms associated-impaired sleep (33), yet this hypothesis has not been well-established. Future studies should be conducted to confirm our results and further elucidate the underlying mechanism.

When combining different allergic symptoms together, our results indicated that the combined effects of multiple allergic symptoms might show stronger associations with higher risk of short sleep duration and irregular sleep among toddlers. The coexistence of different allergic symptoms may represent a systemic allergic disease (e.g., food allergy) or atopic march (increased occurrence of airway allergy after the cutaneous sensitization onset) (9). An UK longitudinal cohort (37) have shown substantially more sleep disturbances in children with both AD and comorbid asthma or AR over 11 years of follow-ups. Another cross-sectional study conducted in the USA (15) indicated that uncontrolled AR might exacerbate asthma, altogether contributing to the increased sleep problems in urban children aged 7–9 years. Our study appeared to be the first to show evidence in linking multiple comorbid allergic symptoms with poor sleep quantity and quality in toddlers, highlighting that screening of allergic symptoms should be conducted as early as possible. It should be noted that allergy in early life is prone to be under-recognized and under-diagnosed due to infantile limited expression ability, poor related knowledge in caregivers, and difficulty in identifying age-unique allergic symptom patterns (7, 8). Therefore, health care professionals and parents must be educated about the damaging influences early allergic symptoms can have on toddlers.

There are several limitations in our study that should be considered. Firstly, we cannot exclude the possibility for recall and misclassification bias of parent-reported allergic symptoms. In order to reduce such misclassification and recall bias, well-trained investigators collected details of age at symptom onset, reproducibility, suspected allergen, and allergy diagnosis, contributing to identifying allergic symptoms more reliably. Secondly, we measured sleep with parent-reported questionnaire, which may have resulted in overestimation of sleep duration and underestimation of sleep disturbances (38). However, questionnaire is highly cost-effective in screening pediatric sleep problems (39), and the BISQ that we adopted has been validated against objective actigraphy (24). Thirdly, for children who had allergic symptoms onset over the past month, the exposure window overlapped with the time of BISQ assessment, which may weaken the association between allergic symptoms and sleep outcomes. However, the proportion of allergic symptoms onset over the past month accounted for 7.92% of symptoms ever happened in this study, suggesting most of the exposure to allergic symptoms had happened before sleep outcomes were assessed. Fourthly, unmeasured confounders (e.g., residential environment) are always a concern in observational studies. Nonetheless, we have included a series of early-life risk factors in the sensitivity analyses, and the associations remained consistent. Finally, although our findings provided clues on the negative impact of allergic symptoms on sleep, we could not examine how the graduation and intensity of allergic symptoms may affect sleep differently, which should be addressed in future study.

## Conclusions

Our study suggested that allergic symptoms in the first 2 years of life were adversely associated with several sleep outcomes. Our findings highlight the importance of strengthening public health efforts to the screening of allergic symptoms in toddlers in early life.

## Data Availability Statement

The datasets presented in this article are not readily available because they are from an ongoing cohort. Requests to access the datasets should be directed to Li Cai, caili5@mail.sysu.edu.cn.

## Ethics Statement

The studies involving human participants were reviewed and approved by the Ethics Committee of the School of Public Health of Sun Yat-sen University. Written informed consent to participate in this study was provided by the participants' legal guardian/next of kin.

## Author Contributions

LC and YC contributed to the conception of the study. YC and LL performed data curation and statistical analysis. YW contributed to results visualization. YC prepared the manuscript draft. LC, LL, and SK critically revised and edited the manuscript. LC and BH supervised the study. LC and JJ obtained funding and material support. WP, SW, and NT performed the investigation. All authors contributed to manuscript revision and approved the final manuscript.

## Funding

This work was supported by the Key-Area Research and Development Program of Guangdong Province (2019B030335001), the “Nutrition and Care of Maternal & Child Research Fund Project” of Biostime Institute of Nutrition & Care (2021BINCMCF053), and the Leadership Improvement Support Program by the Chinese Nutrition Society (CNS2020100B-5).

## Conflict of Interest

The authors declare that the research was conducted in the absence of any commercial or financial relationships that could be construed as a potential conflict of interest.

## Publisher's Note

All claims expressed in this article are solely those of the authors and do not necessarily represent those of their affiliated organizations, or those of the publisher, the editors and the reviewers. Any product that may be evaluated in this article, or claim that may be made by its manufacturer, is not guaranteed or endorsed by the publisher.
