# Supplementary material for: Association of Allergic Symptoms in the First 2 Years of Life With Sleep Outcomes Among Chinese Toddlers
Source: Front Pediatr. 2022 Jan 12;9:791369. doi: 10.3389/fped.2021.791369 (PMC8790483; doi:10.3389/fped.2021.791369)
Supplement: Supplementary file 1 [file Table_1.DOCX]

Supplementary Material

**Supplementary Figure 1 |** The proportion of children with allergic diseases diagnoses by specific allergic symptoms during the first two years of life (*N* = 673)

Any; any allergic symptoms; S: skin symptoms; NO: nasal and ocular symptoms; GI: gastrointestinal symptoms; W: wheeze. There were 37 (5.50%) children ever had mouth and lips symptoms during the first two years of life, among whom 16 were diagnosed as food allergy (data not shown)

Supplementary Table 1 | Sensitivity analysis of adjusted estimates (β) for sleep duration at 2 years of age by allergic symptoms in the first 2 years (*N* = 673)

| Allergic symptoms ^a^ | Total sleep duration  β (95% CI) | | | |  | Nighttime sleep duration  β (95% CI) | | | |  | Daytime sleep duration  β (95% CI) | | | |
| --- | --- | --- | --- | --- | --- | --- | --- | --- | --- | --- | --- | --- | --- | --- |
|  | Main model+ exclusive breastfeeding | Main model  +BMI Z-score | Main model  +preterm birth | Main model  + sleep hygiene |  | Main model+ exclusive breastfeeding | Main model  +BMI Z- score | Main model  + preterm birth | Main model  + sleep hygiene |  | Main model+ exclusive breastfeeding | Main model  +BMI Z- score | Main model  + preterm birth | Main model  + sleep hygiene |
| Skin symptoms |  |  |  |  |  |  |  |  |  |  |  |  |  |  |
| Never had | 0.00 (ref) | 0.00 (ref) | 0.00 (ref) | 0.00 (ref) |  | 0.00 (ref) | 0.00 (ref) | 0.00 (ref) | 0.00 (ref) |  | 0.00 (ref) | 0.00 (ref) | 0.00 (ref) | 0.00 (ref) |
| Ever had | -0.08  (-0.25, 0.08) | -0.07  (-0.23, 0.10) | -0.07  (-0.07, 0.36) | -0.07  (-0.23, 0.10) |  | -0.07  (-0.22, 0.07) | -0.07  (-0.21, 0.07) | -0.07  (-0.21, 0.07) | -0.07  (-0.22, 0.07) |  | -0.01  (-0.11,0.10) | 0.00  (-0.10, 0.10) | 0.00  (-0.10, 0.10) | 0.01  (-0.10, 0.11) |
| Nasal and ocular symptoms |  |  |  |  |  |  |  |  |  |  |  |  |  |  |
| Never had | 0.00 (ref) | 0.00 (ref) | 0.00 (ref) | 0.00 (ref) |  | 0.00 (ref) | 0.00 (ref) | 0.00 (ref) | 0.00 (ref) |  | 0.00 (ref) | 0.00 (ref) | 0.00 (ref) | 0.00 (ref) |
| Ever had | 0.10  (-0.16, 0.37) | 0.10  (-0.17, 0.37) | 0.10  (-0.17, 0.37) | 0.10  (-0.17, 0.37) |  | -0.03  (-0.26, 0.20) | -0.04  (-0.27, 0.19) | -0.04  (-0.27, 0.19) | -0.03  (-0.27, 0.20) |  | 0.13  (-0.03, 0.30) | 0.14  (-0.03, 0.31) | 0.14  (-0.03, 0.31) | 0.13  (-0.04, 0.30) |
| Gastrointestinal symptoms |  |  |  |  |  |  |  |  |  |  |  |  |  |  |
| Never had | 0.00 (ref) | 0.00 (ref) | 0.00 (ref) | 0.00 (ref) |  | 0.00 (ref) | 0.00 (ref) | 0.00 (ref) | 0.00 (ref) |  | 0.00 (ref) | 0.00 (ref) | 0.00 (ref) | 0.00 (ref) |
| Ever had | **-0.27**  **(-0.48, -0.07)** | **-0.27**  **(-0.48, -0.07)** | **-0.28**  **(-0.48, -0.08)** | **-0.28**  **(-0.48, -0.07)** |  | **-0.25**  **(-0.42, -0.07)** | **-0.25**  **(-0.43, -0.07)** | **-0.25**  **(-0.43, -0.08)** | **-0.25**  **(-0.43, -0.08)** |  | -0.03  (-0.16,0.10) | -0.02  (-0.15, 0.11) | -0.03  (-0.16, 0.10) | -0.03  (-0.15, 0.10) |
| Wheeze |  |  |  |  |  |  |  |  |  |  |  |  |  |  |
| Never had | 0.00 (ref) | 0.00 (ref) | 0.00 (ref) | 0.00 (ref) |  | 0.00 (ref) | 0.00 (ref) | 0.00 (ref) | 0.00 (ref) |  | 0.00 (ref) | 0.00 (ref) | 0.00 (ref) | 0.00 (ref) |
| Ever had | 0.13  (-0.15, 0.41) | 0.13  (-0.16, 0.41) | 0.13  (-0.15, 0.00) | -0.03  (-0.16,0.10) |  | 0.01  (-0.23, 0.25) | 0.00  (-0.24, 0.25) | 0.00  (-0.24, 0.24) | 0.00  (-0.24, 0.25) |  | 0.12  (-0.06, 0.30) | 0.12  (-0.06, 0.30) | 0.12  (-0.05, 0.30) | 0.13  (-0.05, 0.30) |
| Allergic multimorbidity |  |  |  |  |  |  |  |  |  |  |  |  |  |  |
| None | 0.00 (ref) | 0.00 (ref) | 0.00 (ref) | 0.00 (ref) |  | 0.00 (ref) | 0.00 (ref) | 0.00 (ref) | 0.00 (ref) |  | 0.00 (ref) | 0.00 (ref) | 0.00 (ref) | 0.00 (ref) |
| Single  symptom | -0.08  (-0.26, 0.10) | -0.07  (-0.25, 0.11) | -0.07  (-0.25, 0.11) | -0.07  (-0.25,0.11) |  | -0.12  (-0.27, 0.04) | -0.11  (-0.27, 0.04) | -0.11  (-0.27, 0.04) | -0.12  (-0.27, 0.04) |  | 0.04  (-0.08, 0.15) | 0.04  (-0.07, 0.16) | 0.04  (-0.07, 0.15) | 0.05  (-0.07, 0.16) |
| Multiple  symptoms | -0.16  (-0.38, 0.06) | -0.16  (-0.38, 0.06) | -0.17  (-0.38, 0.05) | -0.16  (-0.38,0.05) |  | **-0.20**  **(-0.39, -0.01)** | **-0.20**  **(-0.39, -0.02)** | **-0.21**  **(-0.40, -0.02)** | **-0.21**  **(-0.40, -0.02)** |  | 0.04  (-0.10, 0.18) | 0.04  (-0.10, 0.18) | 0.04  (-0.10, 0.18) | 0.05  (-0.09, 0.18) |
| *P* _allergic multimorbidity_ | 0.138 | 0.142 | 0.130 | 0.139 |  | **0.028** | **0.027** | **0.024** | **0.023** |  | 0.503 | 0.478 | 0.488 | 0.44 |

Main model was adjusted for child’s gender, maternal age at enrollment, maternal education level, monthly household income, family history of allergy, delivery mode, household secondhand smoke.

BMI: body mass index; Missing values in covariates were handled by multiple imputation (imputation = 5).

Sleep hygiene factors include how to fall asleep (in bed alone, yes or no) and bed sharing (yes or no).

Statistically significant results (*P* < 0.05) are in bold.

^a^ Mouth and lips symptoms were not significantly associated with total, nighttime, and daytime sleep duration (data not shown).

Supplementary Table 2 | Sensitivity analysis of adjusted odds ratio (OR) for sleep-quality disturbances at 2 years of age by allergic symptoms in the first 2 years (*N* = 673)

| Allergic symptoms ^a^ | Difficulty falling Asleep  OR (95% CI) | | | |  | Frequent nighttime awakening  OR (95% CI) | | | |  | Irregular sleep  OR (95% CI) | | | |
| --- | --- | --- | --- | --- | --- | --- | --- | --- | --- | --- | --- | --- | --- | --- |
|  | Main model+ exclusive breastfeeding | Main model  +BMI Z-score | Main model  +preterm birth | Main model  + sleep hygiene |  | Main model+ exclusive breastfeeding | Main model  +BMI Z-score | Main model  + preterm birth | Main model  + sleep hygiene |  | Main model+ exclusive breastfeeding | Main model  +BMI Z-score | Main model  + preterm birth | Main model  + sleep hygiene |
| Skin symptoms |  |  |  |  |  |  |  |  |  |  |  |  |  |  |
| Never had | 1.00 (ref) | 1.00 (ref) | 1.00 (ref) | 1.00 (ref) |  | 1.00 (ref) | 1.00 (ref) | 1.00 (ref) | 1.00 (ref) |  | 1.00 (ref) | 1.00 (ref) | 1.00 (ref) | 1.00 (ref) |
| Ever had | 1.06  (0.90, 1.25) | 1.05  (0.89, 1.23) | 1.06 (0.90,1.24) | 1.07  (0.91, 1.26) |  | 1.06  (0.85, 1.33) | 1.05  (0.84, 1.32) | 1.05  (0.84, 1.31) | 1.05  (0.84, 1.31) |  | 1.08  (0.86, 1.37) | 1.08  (0.86, 1.37) | 1.08  (0.86, 1.37) | 1.08  (0.85, 1.36) |
| Nasal and ocular symptoms |  |  |  |  |  |  |  |  |  |  |  |  |  |  |
| Never had | 1.00 (ref) | 1.00 (ref) | 1.00 (ref) | 1.00 (ref) |  | 1.00 (ref) | 1.00 (ref) | 1.00 (ref) | 1.00 (ref) |  | 1.00 (ref) | 1.00 (ref) | 1.00 (ref) | 1.00 (ref) |
| Ever had | 1.00  (0.77, 1.31) | 0.99  (0.76, 1.30) | 1.00 (0.77,1.31) | 1.00  (0.76, 1.31) |  | **1.42**  **(1.03, 1.95)** | **1.42**  **(1.04, 1.95**) | **1.41**  **(1.03, 1.94)** | **1.41**  **(1.03, 1.93)** |  | **1.45**  **(1.05, 2.01)** | **1.47**  **(1.06, 2.04)** | **1.46**  **(1.06, 2.03)** | **1.48**  **(1.07, 2.06)** |
| Gastrointestinal symptoms |  |  |  |  |  |  |  |  |  |  |  |  |  |  |
| Never had | 1.00 (ref) | 1.00 (ref) | 1.00 (ref) | 1.00 (ref) |  | 1.00 (ref) | 1.00 (ref) | 1.00 (ref) | 1.00 (ref) |  | 1.00 (ref) | 1.00 (ref) | 1.00 (ref) | 1.00 (ref) |
| Ever had | 1.07  (0.87, 1.31) | 1.07  (0.87, 1.31) | 1.08 (0.88,1.32) | 1.07  (0.87, 1.31) |  | 1.05  (0.80, 1.38) | 1.05  (0.81, 1.38) | 1.06  (0.81, 1.38) | 1.04  (0.80, 1.36) |  | **1.59**  **(1.23, 2.04)** | **1.62**  **(1.26, 2.08)** | **1.62**  **(1.26, 2.10)** | **1.61**  **(1.25, 2.07**) |
| Wheeze |  |  |  |  |  |  |  |  |  |  |  |  |  |  |
| Never had | 1.00 (ref) | 1.00 (ref) | 1.00 (ref) | 1.00 (ref) |  | 1.00 (ref) | 1.00 (ref) | 1.00 (ref) | 1.00 (ref) |  | 1.00 (ref) | 1.00 (ref) | 1.00 (ref) | 1.00 (ref) |
| Ever had | 0.97  (0.73, 1.28) | 0.94  (0.71, 1.24) | 0.97 (0.73,1.28) | 0.96  (0.72, 1.27) |  | 0.95  (0.65, 1.40) | 0.99  (0.68, 1.46) | 0.97  (0.66, 1.42) | 0.96  (0.66, 1.41) |  | 1.27  (0.84, 1.80) | 1.26  (0.88, 1.81) | 1.27  (0.89, 1.80) | 1.25  (0.88, 1.77) |
| Allergic multimorbidity |  |  |  |  |  |  |  |  |  |  |  |  |  |  |
| None | 1.00 (ref) | 1.00 (ref) | 1.00 (ref) | 1.00 (ref) |  | 1.00 (ref) | 1.00 (ref) | 1.00 (ref) | 1.00 (ref) |  | 1.00 (ref) | 1.00 (ref) | 1.00 (ref) | 1.00 (ref) |
| Single symptom | 0.97  (0.68, 1.38) | 0.96  (0.68, 1.37) | 0.96 (0.68,1.37) | 0.99  (0.69, 1.41) |  | 1.25  (0.76, 2.05) | 1.22  (0.74, 2.00) | 1.23  (0.75, 2.02) | 1.21  (0.74, 1.99) |  | 1.55  (0.90, 2.67) | 1.52  (0.89, 2.62) | 1.52  (0.88, 2.62) | 1.52  (0.88, 2.62) |
| Multiple symptoms | 1.32  (0.85, 2.06) | 1.27  (0.82, 1.98) | 1.33 (0.85,2.06) | 1.31  (0.84, 2.03) |  | 1.42  (0.80, 2.53) | 1.46  (0.82, 2.59) | 1.42  (0.80, 2.53) | 1.39  (0.79, 2.46) |  | **2.60**  **(1.44, 4.70)** | **2.58**  **(1.42, 4.67)** | **2.61**  **(1.45, 4.72)** | **2.57**  **(1.43, 4.65)** |
| *P* _allergic multimorbidity_ | 0.304 | 0.378 | 0.295 | 0.303 |  | 0.211 | 0.192 | 0.211 | 0.239 |  | **0.002** | **0.002** | **0.002** | **0.002** |

Main model was adjusted for child’s gender, maternal age at enrollment, maternal education level, monthly household income, family history of allergy, delivery mode, household secondhand smoke.

BMI: body mass index; Missing values in covariates were handled by multiple imputation (imputation = 5).

Sleep hygiene factors include how to fall asleep (in bed alone, yes or no) and bed sharing (yes or no).

Statistically significant results (*P* < 0.05) are in bold.

^a^ Mouth and lips symptoms were not significantly associated with difficulty falling asleep, frequent nighttime awakening, and irregular sleep (data not shown).

Supplementary Table 3 | Logistic regression of allergic symptoms in the first 2 years with insufficient sleep at 2 years of age (*N* = 662)

| Allergic symptoms ^a^ | Insufficient sleep [OR (95% CI)] | | | | |
| --- | --- | --- | --- | --- | --- |
|  | Main model | Main model+ exclusive breastfeeding | Main model  +BMI Z-score | Main model  +preterm birth | Main model  +sleep hygiene |
| Skin symptoms |  |  |  |  |  |
| Never had | 1.00 (ref) | 1.00 (ref) | 1.00 (ref) | 1.00 (ref) | 1.00 (ref) |
| Ever had | 1.03 (0.94, 1.40) | 1.16 (0.95, 1.42) | 1.15 (0.94, 1.41) | 1.14 (0.93,1.40) | 1.14 (0.93, 1.39) |
| Nasal and ocular symptoms |  |  |  |  |  |
| Never had | 1.00 (ref) | 1.00 (ref) | 1.00 (ref) | 1.00 (ref) | 1.00 (ref) |
| Ever had | 0.87 (0.61, 1.25) | 0.87 (0.61, 1.25) | 0.86 (0.60, 1.24) | 0.86 (0.60, 1.24) | 0.86 (0.60, 1.23) |
| Gastrointestinal symptoms |  |  |  |  |  |
| Never had | 1.00 (ref) | 1.00 (ref) | 1.00 (ref) | 1.00 (ref) | 1.00 (ref) |
| Ever had | **1.27 (1.01, 1.61)** | **1.28 (1.01, 1.61)** | **1.28 (1.01, 1.62)** | **1.27 (1.01, 1.61)** | **1.26 (1.00, 1.60)** |
| Wheeze |  |  |  |  |  |
| Never had | 1.00 (ref) | 1.00 (ref) | 1.00 (ref) | 1.00 (ref) | 1.00 (ref) |
| Ever had | 0.89 (0.61, 1.30) | 0.88 (0.61, 1.29) | 0.91 (0.62, 1.33) | 0.89 (0.61, 1.30) | 0.88 (0.60,1.29) |
| Allergic multimorbidity |  |  |  |  |  |
| None | 1.00 (ref) | 1.00 (ref) | 1.00 (ref) | 1.00 (ref) | 1.00 (ref) |
| Single symptom | **1.57 (1.00, 2.46)** | **1.60 (1.02, 2.51)** | **1.57 (1.00, 2.46)** | **1.57 (1.00, 2.46)** | 1.54 (0.98, 2.41) |
| Multiple symptoms | 1.49 (0.87, 2.55) | 1.53 (0.89, 2.64) | 1.52 (0.88, 2.61) | 1.48 (0.86, 2.54) | 1.44 (0.84, 2.47) |
| *P* _allergic multimorbidity_ | 0.086 | 0.068 | 0.091 | 0.093 | 0.109 |

Insufficient sleep: total sleep duration < 11h; total sleep duration > 14h were excluded in the analysis (n = 11).

Main model was adjusted for child’s gender, maternal age at enrollment, maternal education level, monthly household income, family history of allergy, delivery mode, household secondhand smoke.

BMI: body mass index. Missing values in covariates were handled by multiple imputation (imputation = 5).

Sleep hygiene factors include how to fall asleep (in bed alone, yes or no) and bed sharing (yes or no).

Statistically significant results (*P* < 0.05) are in bold.

^a^ Mouth and lips symptoms were not significantly associated with insufficient sleep (data not shown).

Supplementary Table 4 | Logistic regression of allergic symptoms in the first 2 years with difficulty falling asleep (SOL > 30 min) at 2 years of age (*N* = 673)

| Allergic symptoms ^a^ | Difficulty falling asleep [OR (95% CI)] | | | | |
| --- | --- | --- | --- | --- | --- |
|  | Main model | Main model+ exclusive breastfeeding | Main model  +BMI Z-score | Main model  +preterm birth | Main model  +sleep hygiene |
| Skin symptoms |  |  |  |  |  |
| Never had | 1.00 (ref) | 1.00 (ref) | 1.00 (ref) | 1.00 (ref) | 1.00 (ref) |
| Ever had | 1.01 (0.82, 1.25) | 1.02 (0.83, 1.26) | 1.03 (1.06, 1.27) | 1.01 (0.82, 1.25) | 1.03 (0.83, 1.27) |
| Nasal and ocular symptoms |  |  |  |  |  |
| Never had | 1.00 (ref) | 1.00 (ref) | 1.00 (ref) | 1.00 (ref) | 1.00 (ref) |
| Ever had | 0.95 (0.67, 1.35) | 0.95 (0.67, 1.36) | 0.93 (0.66, 1.33) | 0.94 (0.66, 1.33) | 0.93 (0.66, 1.32) |
| Gastrointestinal symptoms |  |  |  |  |  |
| Never had | 1.00 (ref) | 1.00 (ref) | 1.00 (ref) | 1.00 (ref) | 1.00 (ref) |
| Ever had | 1.27 (0.99, 1.62) | 1.27 (0.99, 1.62) | 1.27 (1.00, 1.63) | 1.26 (0.99, 1.61) | 1.25 (0.97, 1.59) |
| Wheeze |  |  |  |  |  |
| Never had | 1.00 (ref) | 1.00 (ref) | 1.00 (ref) | 1.00 (ref) | 1.00 (ref) |
| Ever had | 0.85 (0.57, 1.26) | 0.85 (0.57, 1.27) | 0.86 (0.58, 1.28) | 0.84 (0.57, 1.25) | 0.82 (0.55, 1.22) |
| Allergic multimorbidity |  |  |  |  |  |
| None | 1.00 (ref) | 1.00 (ref) | 1.00 (ref) | 1.00 (ref) | 1.00 (ref) |
| Single symptom | 1.04 (0.65,1.66) | 1.04 (0.65,1.67) | 1.05 (0.66, 1.68) | 1.04 (0.65,1.66) | 1.04 (0.65, 1.66) |
| Multiple symptoms | 1.12 (0.65,1.93) | 1.12 (0.65,1.95) | 1.14 (0.66, 1.98) | 1.07 (0.62,1.86) | 1.09 (0.63, 1.89) |
| *P* _allergic multimorbidity_ | 0.699 | 0.682 | 0.638 | 0.799 | 0.753 |

Difficulty falling asleep: sleep onset latency (SOL) > 30 min.

Main model was adjusted for child’s gender, maternal age at enrollment, maternal education level, monthly household income, family history of allergy, delivery mode, household secondhand smoke.

BMI: body mass index. Missing values in covariates were handled by multiple imputation (imputation = 5).

Sleep hygiene factors include how to fall asleep (in bed alone, yes or no) and bed sharing (yes or no).

Statistically significant results (*P* < 0.05) are in bold.

^a^ Mouth and lips symptoms were not significantly associated with difficulty falling asleep (data not shown).
