# Supplementary figures and images for: Association of Allergic Symptoms in the First 2 Years of Life With Sleep Outcomes Among Chinese Toddlers
Source: Front Pediatr. 2022 Jan 12;9:791369. doi: 10.3389/fped.2021.791369 (PMC8790483; doi:10.3389/fped.2021.791369)

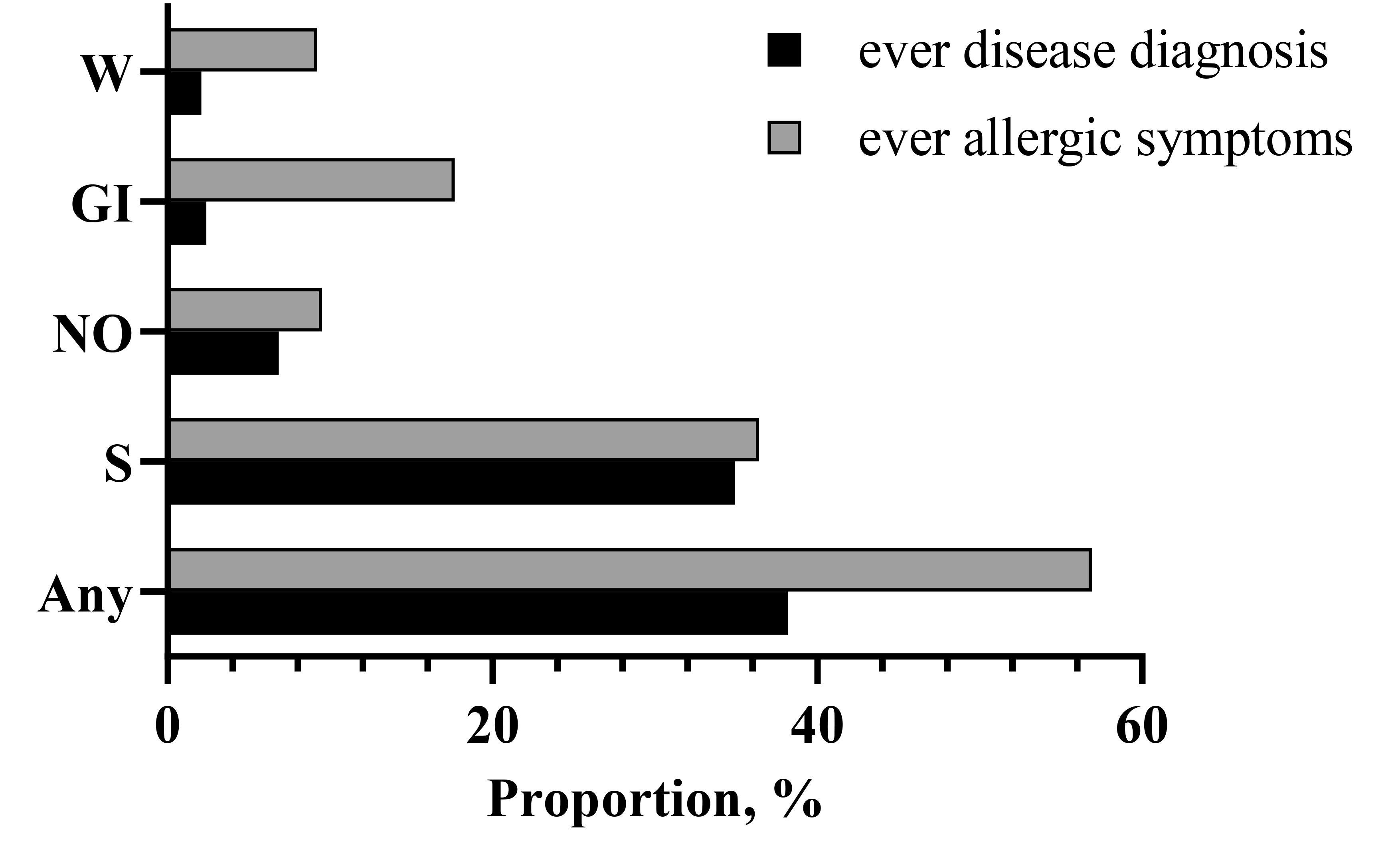

Supplement: Supplementary file 2 [file Image_1.TIF]
